# (Carbonato-κ^2^ *O*,*O*′)bis­(5,5′-dimethyl-2,2′-bipyridyl-κ^2^ *N*,*N*′)cobalt(III) bromide trihydrate

**DOI:** 10.1107/S160053681200894X

**Published:** 2012-03-07

**Authors:** Kannan Arun Kumar, Parthsarathi Meera, Madhavan Amutha Selvi, Arunachalam Dayalan

**Affiliations:** a Department of Chemistry, Loyola College (Autonomous), Chennai 600 034, India

## Abstract

In the title complex, [Co(CO_3_)(C_12_H_12_N_2_)_2_]Br·3H_2_O, the Co^III^ cation has a distorted octa­hedral coordination environment. It is chelated by four N atoms of two different 5,5′-dimethyl-2,2′-bipyridyl (dmbpy) ligands in axial and equatorial positions, and by two O atoms of a carbonate anion completing the equatorial positions. Although the water mol­ecules are disordered and their H atoms were not located, there are typical O⋯O distances between 2.8 and 3.0 Å, indicating O—H⋯O hydrogen bonding. The crystal packing is consolidated by C—H⋯O and C—H⋯Br hydrogen bonds, as well as π–π stacking inter­actions between adjacent pyridine rings of the dmbpy ligands, with centroid–centroid distances of 3.694 (3) and 3.7053 (3) Å.

## Related literature

For background to this class of compounds, see: Momeni *et al.* (2009 ▶); Harding *et al.* (2008 ▶); Kusrini *et al.* (2008 ▶). For applications of this class of compounds in various fields, see: Carol *et al.* (2006 ▶); Eddie *et al.* (2010 ▶); Raj *et al.* (2008 ▶); Vitushkina *et al.* (2006 ▶); Hyung *et al.* (2006 ▶); Jayaweera *et al.* (2002 ▶), Shi *et al.* (2010 ▶); For similar structures, see: Ma *et al.* (2008 ▶); Phatchimkun & Chaichit (2011 ▶).
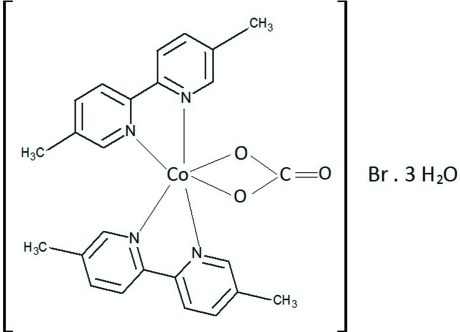


## Experimental

### 

#### Crystal data

[Co(CO_3_)(C_12_H_12_N_2_)_2_]Br·3H_2_O
*M* *_r_* = 621.32
Monoclinic, 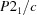

*a* = 11.5802 (15) Å
*b* = 15.958 (2) Å
*c* = 14.3921 (17) Å
β = 100.143 (3)°
*V* = 2618.0 (6) Å^3^
*Z* = 4
Mo *K*α radiation
μ = 2.23 mm^−1^
*T* = 293 K
0.30 × 0.25 × 0.20 mm

#### Data collection

Bruker APEXII CCD diffractometer
Absorption correction: multi-scan (*SADABS*; Bruker, 2004 ▶) *T*_min_ = 0.555, *T*_max_ = 0.664
23769 measured reflections
5033 independent reflections
3111 reflections with *I* > 2σ(*I*)
*R*_int_ = 0.048

#### Refinement

*R*[*F* ^2^ > 2σ(*F* ^2^)] = 0.046
*wR*(*F* ^2^) = 0.145
*S* = 1.02
5033 reflections
346 parameters
H-atom parameters constrained
Δρ_max_ = 0.77 e Å^−3^
Δρ_min_ = −1.43 e Å^−3^

### 

Data collection: *APEX2* (Bruker, 2004 ▶); cell refinement: *SAINT* (Bruker, 2004 ▶); data reduction: *SAINT*; program(s) used to solve structure: *SIR92* (Altomare *et al.*, 1993 ▶); program(s) used to refine structure: *SHELXL97* (Sheldrick, 2008 ▶); molecular graphics: *ORTEP-3* (Farrugia, 1997 ▶) and *Mercury* (Macrae *et al.*, 2008 ▶); software used to prepare material for publication: *publCIF* (Westrip, 2010 ▶).

## Supplementary Material

Crystal structure: contains datablock(s) I, global. DOI: 10.1107/S160053681200894X/wm2590sup1.cif

Structure factors: contains datablock(s) I. DOI: 10.1107/S160053681200894X/wm2590Isup2.hkl

Additional supplementary materials:  crystallographic information; 3D view; checkCIF report

## Figures and Tables

**Table 1:** Hydrogen-bond geometry (Å, °)

*D*—H⋯*A*	*D*—H	H⋯*A*	*D*⋯*A*	*D*—H⋯*A*
C1—H1⋯O1	0.93	2.43	2.931 (5)	114
C3—H3⋯Br1^i^	0.93	2.80	3.718 (4)	168
C4—H4⋯O1^ii^	0.93	2.51	3.257 (4)	138
C11—H11*A*⋯Br1^iii^	0.96	2.91	3.810 (4)	157
C19—H19⋯O3^iv^	0.93	2.52	3.284 (4)	140
C20—H20⋯Br1^iv^	0.93	2.85	3.778 (4)	172
C22—H22⋯O3	0.93	2.44	2.939 (4)	113
C23—H23*B*⋯O4^v^	0.96	2.42	3.330 (6)	158
C24—H24*A*⋯Br1	0.96	2.93	3.836 (5)	158

Symmetry codes: (i) 
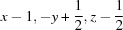
; (ii) 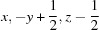; (iii) 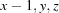; (iv) 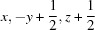; (v) 
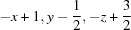
.

**Fig. 1.**
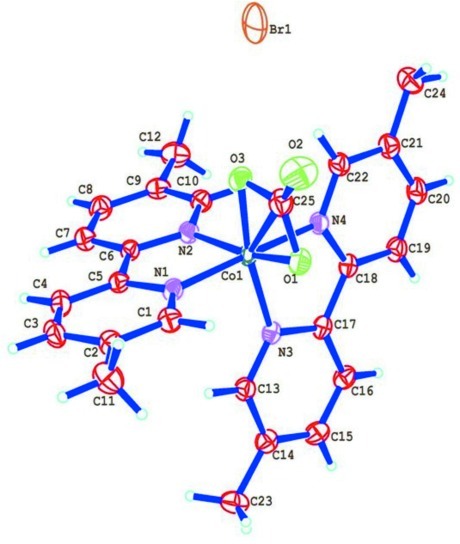
Ellipsoid plot (50% probability) of the cationic complex and the bromide anion. Disordered water molecules were omitted for clarity.

**Fig. 2.**
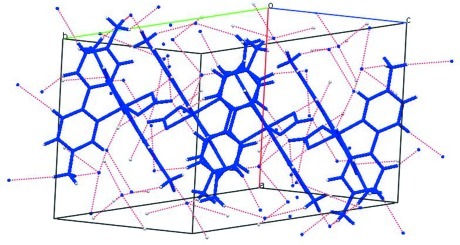
Crystal packing of the complex, showing also the hydrogen bonding interactions (dotted lines)

## supplementary crystallographic information

### Comment

2,2'-Bipyridyl and 1,10-phenanthroline are versatile ligands capable of forming stable complexes with different metals in their various oxidation states (Momeni *et al.*, 2009; Harding *et al.*, 2008; Kusrini *et al.*, 2008; Phatchimkun & Chaichit, 2011; Ma *et al.*, 2008). These complexes have interesting electrical, magnetic, thermal, optical and antimicrobial properties. Hence, they are widely studied in various fields like medicine (Carol *et al.*, 2006; Eddie *et al.*, 2010), crystallography (Raj *et al.*, 2008; Vitushkina *et al.*,2006;) chemistry (Hyung *et al.*, 2006; Jayaweera *et al.*, 2002) or biology (Shi *et al.*, 2010). The crystal structures of a large number of metal complexes with the above mentioned ligands have been reported, including substituted ligands with various moieties like halogens, methyl, phenyl, acetyl at various positions.

The title complex [Co(C_12_H_12_N_2_)_2_CO_3_]Br^.^3H_2_O, consists of a complex cation [Co(C_12_H_12_N_2_)_2_CO_3_]^+^, a bromide counter anion and three molecules of lattice water. The cobalt(III) ion is six coordinated by four nitrogen atoms of the two 5,5'-dimethyl-2,2'-bipyridyl (dmbpy) ligands and two oxygen atoms of the carbonato ligands in a distorted octahedral environment (Fig. 1). The water molecules are disordered in the crystal packing, but O···O distances indicate O—H···O hydrogen bonding. The dihedral angle between the two dmbpy ligands is 85.8 (2)°. The major distortion from the ideal geometry is due to a narrow bite angle of the carbonato ligand, *i.e.* O1–Co—O3 = 69.44 (11)°. The bromide ion and solvated water molecules are dispersed between cationic layers. The crystal packing is stabilized by extensive series of C—H···O and C—H···Br hydrogen bonds (Fig.2, Table.1) In addition, π—π stacking interactions between adjacent pyridine rings are present with centroid to centroid distances of 3.694 (3) A° and 3.7053 (3) A°.

### Experimental

The title complex was prepared by mixing 0.005 mol of finely crushed cobalt(II) bromide, exposed to microwave radiation and dissolved in 75 ml of acetone, with 0.005 mol of sodium carbonate, 0.01 mol of dmbpy and 2 ml hydrogen peroxide (30% *v*/*v*). The reaction mixture was stirred and allowed to react for one hour. The resultant pink coloured precipitate was filtered, washed with excess acetone and dried over vacuum desicator (yield: 60%). Dark red coloured single crystals were grown from a 90% *v*/*v* ethanolic solution by slow evoparation.

### Refinement

The highest difference electron density is 0.77 e/Å ^3^ and is located at a distance of 0.902 Å away from bromine atom. The deepest hole is -1.43 e/Å ^3^ and is located at a distance of 0.83 Å a away from bromine atom. The solvent water molecules are disordered over various sites. Their occupancy was constrained for unity. Hydrogen atoms of the water molecules could not be reliably located in difference Fourier maps and hence were excluded from the refinement.

### Crystal data

**Table:**

[Co(CO_3_)(C_12_H_12_N_2_)_2_]Br·3H_2_O	*F*(000) = 1248
*M**_r_* = 621.32	*D*_x_ = 1.561 Mg m^−^^3^
Monoclinic, *P*2_1_/*c*	Mo *K*α radiation, λ = 0.71073 Å
Hall symbol: -P 2ybc	Cell parameters from 4593 reflections
*a* = 11.5802 (15) Å	θ = 2.2–25.6°
*b* = 15.958 (2) Å	µ = 2.23 mm^−^^1^
*c* = 14.3921 (17) Å	*T* = 293 K
β = 100.143 (3)°	Block, red
*V* = 2618.0 (6) Å^3^	0.30 × 0.25 × 0.20 mm
*Z* = 4	

### Data collection

**Table:**

Bruker APEXII CCD diffractometer	5033 independent reflections
Radiation source: fine-focus sealed tube	3111 reflections with *I* > 2σ(*I*)
Graphite monochromator	*R*_int_ = 0.048
ω and φ scan	θ_max_ = 25.9°, θ_min_ = 2.2°
Absorption correction: multi-scan (*SADABS*; Bruker, 2004)	*h* = −14→14
*T*_min_ = 0.555, *T*_max_ = 0.664	*k* = −19→19
23769 measured reflections	*l* = −17→17

### Refinement

**Table:**

Refinement on *F*^2^	Primary atom site location: structure-invariant direct methods
Least-squares matrix: full	Secondary atom site location: difference Fourier map
*R*[*F*^2^ > 2σ(*F*^2^)] = 0.046	Hydrogen site location: inferred from neighbouring sites
*wR*(*F*^2^) = 0.145	H-atom parameters constrained
*S* = 1.02	*w* = 1/[σ^2^(*F*_o_^2^) + (0.0686*P*)^2^ + 2.4848*P*] where *P* = (*F*_o_^2^ + 2*F*_c_^2^)/3
5033 reflections	(Δ/σ)_max_ < 0.001
346 parameters	Δρ_max_ = 0.77 e Å^−^^3^
0 restraints	Δρ_min_ = −1.43 e Å^−^^3^

### Special details

**Table:**

Geometry. All e.s.d.'s (except the e.s.d. in the dihedral angle between two l.s. planes) are estimated using the full covariance matrix. The cell e.s.d.'s are taken into account individually in the estimation of e.s.d.'s in distances, angles and torsion angles; correlations between e.s.d.'s in cell parameters are only used when they are defined by crystal symmetry. An approximate (isotropic) treatment of cell e.s.d.'s is used for estimating e.s.d.'s involving l.s. planes.
Refinement. Refinement of *F*^2^ against ALL reflections. The weighted *R*-factor *wR* and goodness of fit *S* are based on *F*^2^, conventional *R*-factors *R* are based on *F*, with *F* set to zero for negative *F*^2^. The threshold expression of *F*^2^ > σ(*F*^2^) is used only for calculating *R*-factors(gt) *etc*. and is not relevant to the choice of reflections for refinement. *R*-factors based on *F*^2^ are statistically about twice as large as those based on *F*, and *R*- factors based on ALL data will be even larger.

### Fractional atomic coordinates and isotropic or equivalent isotropic displacement parameters (Å^2^)

**Table:**

	*x*	*y*	*z*	*U*_iso_*/*U*_eq_	Occ. (<1)
C1	0.3712 (3)	0.2890 (2)	0.7720 (3)	0.0345 (8)	
H1	0.3549	0.3252	0.8186	0.041*	
C2	0.2916 (3)	0.2835 (2)	0.6885 (3)	0.0360 (9)	
C3	0.3182 (4)	0.2288 (2)	0.6203 (3)	0.0402 (10)	
H3	0.2675	0.2237	0.5627	0.048*	
C4	0.4194 (3)	0.1824 (2)	0.6379 (2)	0.0374 (9)	
H4	0.4366	0.1450	0.5926	0.045*	
C5	0.4953 (3)	0.1909 (2)	0.7222 (2)	0.0301 (8)	
C6	0.6068 (3)	0.1471 (2)	0.7492 (2)	0.0300 (8)	
C7	0.6501 (3)	0.0889 (2)	0.6939 (3)	0.0389 (9)	
H7	0.6063	0.0731	0.6360	0.047*	
C8	0.7597 (3)	0.0539 (2)	0.7250 (3)	0.0406 (9)	
H8	0.7893	0.0140	0.6884	0.049*	
C9	0.8246 (3)	0.0785 (2)	0.8104 (3)	0.0372 (9)	
C10	0.7749 (3)	0.1367 (2)	0.8627 (2)	0.0342 (8)	
H10	0.8176	0.1539	0.9203	0.041*	
C11	0.1830 (3)	0.3362 (3)	0.6723 (3)	0.0497 (11)	
H11A	0.1417	0.3292	0.7241	0.074*	
H11B	0.1334	0.3193	0.6148	0.074*	
H11C	0.2041	0.3940	0.6678	0.074*	
C12	0.9454 (4)	0.0463 (3)	0.8466 (3)	0.0533 (11)	
H12A	1.0005	0.0917	0.8510	0.080*	
H12B	0.9654	0.0045	0.8041	0.080*	
H12C	0.9478	0.0219	0.9079	0.080*	
C13	0.4108 (3)	0.1317 (2)	0.9421 (2)	0.0337 (8)	
H13	0.3688	0.1440	0.8825	0.040*	
C14	0.3629 (3)	0.0754 (2)	0.9986 (3)	0.0344 (8)	
C15	0.4271 (3)	0.0582 (2)	1.0863 (3)	0.0382 (9)	
H15	0.3983	0.0201	1.1255	0.046*	
C16	0.5334 (3)	0.0966 (2)	1.1166 (2)	0.0346 (8)	
H16	0.5761	0.0852	1.1763	0.042*	
C17	0.5761 (3)	0.1526 (2)	1.0571 (2)	0.0294 (8)	
C18	0.6864 (3)	0.1986 (2)	1.0812 (2)	0.0298 (8)	
C19	0.7652 (3)	0.1912 (2)	1.1642 (2)	0.0373 (9)	
H19	0.7504	0.1541	1.2105	0.045*	
C20	0.8656 (3)	0.2384 (2)	1.1787 (3)	0.0387 (9)	
H20	0.9190	0.2335	1.2348	0.046*	
C21	0.8874 (3)	0.2938 (2)	1.1092 (2)	0.0369 (9)	
C22	0.8051 (3)	0.2976 (2)	1.0263 (2)	0.0350 (9)	
H22	0.8187	0.3337	0.9787	0.042*	
C23	0.2454 (3)	0.0377 (3)	0.9622 (3)	0.0474 (10)	
H23A	0.2341	−0.0107	0.9992	0.071*	
H23B	0.2414	0.0215	0.8975	0.071*	
H23C	0.1852	0.0781	0.9665	0.071*	
C24	0.9940 (3)	0.3485 (3)	1.1216 (3)	0.0508 (11)	
H24A	1.0361	0.3387	1.0709	0.076*	
H24B	1.0436	0.3356	1.1806	0.076*	
H24C	0.9706	0.4062	1.1214	0.076*	
N1	0.4700 (2)	0.24492 (18)	0.78871 (19)	0.0301 (7)	
N2	0.6678 (2)	0.16936 (18)	0.83404 (19)	0.0313 (7)	
N3	0.5147 (2)	0.16874 (18)	0.96982 (18)	0.0294 (7)	
N4	0.7072 (3)	0.25148 (17)	1.01256 (19)	0.0304 (6)	
C25	0.5843 (3)	0.3968 (2)	0.8970 (3)	0.0398 (9)	
Co1	0.58872 (4)	0.25212 (3)	0.90051 (3)	0.02805 (16)	
O1	0.5219 (2)	0.34952 (16)	0.94414 (17)	0.0381 (6)	
O2	0.5824 (3)	0.47344 (18)	0.8941 (2)	0.0651 (9)	
O3	0.6496 (2)	0.34948 (15)	0.85188 (16)	0.0365 (6)	
Br1	1.09456 (5)	0.26057 (4)	0.90092 (4)	0.0860 (2)	
O4	0.7273 (5)	0.5300 (3)	0.7641 (3)	0.1209 (16)	
O5	0.9316 (14)	0.4389 (5)	0.8542 (6)	0.140 (4)	0.80 (2)
O5'	1.012 (5)	0.436 (2)	0.869 (3)	0.140 (4)	0.20 (2)
O6	0.8141 (11)	0.5262 (7)	1.0104 (8)	0.158 (5)	0.761 (18)
O6'	0.745 (3)	0.560 (2)	1.046 (3)	0.158 (5)	0.239 (18)

### Atomic displacement parameters (Å^2^)

**Table:**

	*U*^11^	*U*^22^	*U*^33^	*U*^12^	*U*^13^	*U*^23^
C1	0.039 (2)	0.031 (2)	0.033 (2)	0.0002 (17)	0.0044 (16)	0.0027 (16)
C2	0.040 (2)	0.034 (2)	0.032 (2)	−0.0010 (17)	−0.0007 (16)	0.0069 (16)
C3	0.044 (2)	0.043 (2)	0.029 (2)	−0.0060 (18)	−0.0069 (16)	0.0008 (16)
C4	0.045 (2)	0.039 (2)	0.0268 (19)	−0.0022 (18)	0.0016 (16)	−0.0030 (16)
C5	0.037 (2)	0.0279 (19)	0.0262 (18)	−0.0059 (16)	0.0075 (15)	0.0007 (14)
C6	0.037 (2)	0.0311 (19)	0.0220 (18)	−0.0066 (16)	0.0049 (14)	0.0014 (14)
C7	0.044 (2)	0.039 (2)	0.033 (2)	−0.0071 (18)	0.0070 (16)	−0.0048 (17)
C8	0.046 (2)	0.038 (2)	0.040 (2)	0.0014 (18)	0.0135 (18)	−0.0051 (17)
C9	0.035 (2)	0.035 (2)	0.042 (2)	−0.0011 (17)	0.0090 (17)	0.0055 (17)
C10	0.038 (2)	0.034 (2)	0.0287 (19)	−0.0032 (17)	0.0016 (15)	0.0057 (15)
C11	0.043 (2)	0.049 (3)	0.052 (3)	0.006 (2)	−0.0060 (19)	0.004 (2)
C12	0.041 (2)	0.055 (3)	0.064 (3)	0.004 (2)	0.009 (2)	0.003 (2)
C13	0.038 (2)	0.035 (2)	0.0273 (19)	−0.0020 (17)	0.0049 (15)	0.0004 (15)
C14	0.038 (2)	0.0292 (19)	0.037 (2)	0.0040 (16)	0.0106 (16)	−0.0027 (16)
C15	0.042 (2)	0.035 (2)	0.040 (2)	0.0020 (17)	0.0137 (18)	0.0056 (16)
C16	0.039 (2)	0.038 (2)	0.0273 (19)	0.0040 (17)	0.0057 (15)	0.0096 (15)
C17	0.036 (2)	0.0291 (19)	0.0234 (18)	0.0054 (16)	0.0057 (14)	0.0016 (14)
C18	0.036 (2)	0.031 (2)	0.0222 (17)	0.0027 (16)	0.0039 (14)	0.0000 (14)
C19	0.040 (2)	0.044 (2)	0.0263 (19)	0.0027 (18)	0.0026 (15)	0.0016 (16)
C20	0.039 (2)	0.047 (2)	0.027 (2)	0.0045 (19)	−0.0020 (16)	−0.0030 (17)
C21	0.037 (2)	0.038 (2)	0.035 (2)	0.0012 (17)	0.0043 (16)	−0.0079 (17)
C22	0.041 (2)	0.035 (2)	0.028 (2)	−0.0053 (18)	0.0032 (15)	0.0011 (15)
C23	0.040 (2)	0.046 (2)	0.057 (3)	−0.0114 (19)	0.0104 (19)	0.000 (2)
C24	0.044 (2)	0.057 (3)	0.050 (3)	−0.009 (2)	0.0020 (19)	−0.009 (2)
N1	0.0346 (16)	0.0322 (16)	0.0231 (15)	−0.0002 (14)	0.0044 (12)	0.0045 (12)
N2	0.0353 (17)	0.0325 (17)	0.0256 (15)	−0.0027 (14)	0.0035 (12)	0.0018 (12)
N3	0.0345 (17)	0.0310 (16)	0.0229 (15)	0.0016 (13)	0.0053 (12)	0.0015 (12)
N4	0.0379 (17)	0.0295 (15)	0.0233 (15)	−0.0034 (14)	0.0040 (12)	−0.0011 (12)
C25	0.051 (2)	0.034 (2)	0.033 (2)	−0.001 (2)	0.0039 (17)	0.0021 (18)
Co1	0.0354 (3)	0.0294 (3)	0.0184 (3)	−0.0015 (2)	0.00190 (18)	0.0018 (2)
O1	0.0463 (16)	0.0376 (15)	0.0312 (14)	0.0005 (12)	0.0087 (11)	−0.0018 (11)
O2	0.082 (2)	0.0318 (17)	0.086 (2)	0.0046 (16)	0.0279 (19)	0.0062 (16)
O3	0.0474 (16)	0.0339 (14)	0.0289 (14)	−0.0032 (12)	0.0084 (11)	0.0051 (11)
Br1	0.0908 (5)	0.1163 (6)	0.0404 (3)	0.0077 (4)	−0.0173 (3)	−0.0045 (3)
O4	0.181 (5)	0.098 (3)	0.093 (3)	−0.033 (3)	0.049 (3)	−0.006 (3)
O5	0.149 (11)	0.099 (4)	0.165 (6)	−0.033 (6)	0.005 (7)	0.029 (4)
O5'	0.149 (11)	0.099 (4)	0.165 (6)	−0.033 (6)	0.005 (7)	0.029 (4)
O6	0.118 (8)	0.152 (8)	0.184 (8)	−0.041 (7)	−0.032 (7)	0.093 (7)
O6'	0.118 (8)	0.152 (8)	0.184 (8)	−0.041 (7)	−0.032 (7)	0.093 (7)

### Geometric parameters (Å, º)

**Table:**

C1—N1	1.328 (4)	C15—C16	1.376 (5)
C1—C2	1.383 (5)	C15—H15	0.9300
C1—H1	0.9300	C16—C17	1.388 (5)
C2—C3	1.387 (5)	C16—H16	0.9300
C2—C11	1.497 (5)	C17—N3	1.355 (4)
C3—C4	1.372 (5)	C17—C18	1.461 (5)
C3—H3	0.9300	C18—N4	1.353 (4)
C4—C5	1.375 (5)	C18—C19	1.374 (5)
C4—H4	0.9300	C19—C20	1.371 (5)
C5—N1	1.358 (4)	C19—H19	0.9300
C5—C6	1.459 (5)	C20—C21	1.390 (5)
C6—N2	1.346 (4)	C20—H20	0.9300
C6—C7	1.374 (5)	C21—C22	1.391 (5)
C7—C8	1.386 (5)	C21—C24	1.496 (5)
C7—H7	0.9300	C22—N4	1.337 (4)
C8—C9	1.381 (5)	C22—H22	0.9300
C8—H8	0.9300	C23—H23A	0.9600
C9—C10	1.382 (5)	C23—H23B	0.9600
C9—C12	1.495 (5)	C23—H23C	0.9600
C10—N2	1.341 (4)	C24—H24A	0.9600
C10—H10	0.9300	C24—H24B	0.9600
C11—H11A	0.9600	C24—H24C	0.9600
C11—H11B	0.9600	N1—Co1	1.927 (3)
C11—H11C	0.9600	N2—Co1	1.951 (3)
C12—H12A	0.9600	N3—Co1	1.950 (3)
C12—H12B	0.9600	N4—Co1	1.925 (3)
C12—H12C	0.9600	C25—O2	1.225 (5)
C13—N3	1.337 (4)	C25—O1	1.312 (4)
C13—C14	1.391 (5)	C25—O3	1.318 (4)
C13—H13	0.9300	C25—Co1	2.309 (4)
C14—C15	1.374 (5)	Co1—O3	1.891 (2)
C14—C23	1.496 (5)	Co1—O1	1.892 (2)
			
N1—C1—C2	123.0 (3)	C19—C20—C21	119.8 (3)
N1—C1—H1	118.5	C19—C20—H20	120.1
C2—C1—H1	118.5	C21—C20—H20	120.1
C1—C2—C3	117.3 (3)	C20—C21—C22	117.4 (3)
C1—C2—C11	120.8 (4)	C20—C21—C24	122.3 (3)
C3—C2—C11	121.9 (3)	C22—C21—C24	120.2 (4)
C4—C3—C2	119.8 (3)	N4—C22—C21	122.6 (3)
C4—C3—H3	120.1	N4—C22—H22	118.7
C2—C3—H3	120.1	C21—C22—H22	118.7
C3—C4—C5	120.2 (4)	C14—C23—H23A	109.5
C3—C4—H4	119.9	C14—C23—H23B	109.5
C5—C4—H4	119.9	H23A—C23—H23B	109.5
N1—C5—C4	120.0 (3)	C14—C23—H23C	109.5
N1—C5—C6	114.0 (3)	H23A—C23—H23C	109.5
C4—C5—C6	125.9 (3)	H23B—C23—H23C	109.5
N2—C6—C7	121.1 (3)	C21—C24—H24A	109.5
N2—C6—C5	114.4 (3)	C21—C24—H24B	109.5
C7—C6—C5	124.5 (3)	H24A—C24—H24B	109.5
C6—C7—C8	119.5 (3)	C21—C24—H24C	109.5
C6—C7—H7	120.3	H24A—C24—H24C	109.5
C8—C7—H7	120.3	H24B—C24—H24C	109.5
C9—C8—C7	119.8 (4)	C1—N1—C5	119.7 (3)
C9—C8—H8	120.1	C1—N1—Co1	125.8 (2)
C7—C8—H8	120.1	C5—N1—Co1	114.5 (2)
C8—C9—C10	117.5 (3)	C10—N2—C6	119.0 (3)
C8—C9—C12	122.8 (4)	C10—N2—Co1	126.9 (2)
C10—C9—C12	119.7 (4)	C6—N2—Co1	114.0 (2)
N2—C10—C9	123.0 (3)	C13—N3—C17	119.4 (3)
N2—C10—H10	118.5	C13—N3—Co1	127.1 (2)
C9—C10—H10	118.5	C17—N3—Co1	113.4 (2)
C2—C11—H11A	109.5	C22—N4—C18	119.4 (3)
C2—C11—H11B	109.5	C22—N4—Co1	125.6 (2)
H11A—C11—H11B	109.5	C18—N4—Co1	115.0 (2)
C2—C11—H11C	109.5	O2—C25—O1	125.7 (4)
H11A—C11—H11C	109.5	O2—C25—O3	124.3 (4)
H11B—C11—H11C	109.5	O1—C25—O3	110.0 (3)
C9—C12—H12A	109.5	O2—C25—Co1	179.2 (3)
C9—C12—H12B	109.5	O1—C25—Co1	55.03 (18)
H12A—C12—H12B	109.5	O3—C25—Co1	54.97 (17)
C9—C12—H12C	109.5	O3—Co1—O1	69.44 (11)
H12A—C12—H12C	109.5	O3—Co1—N4	93.28 (11)
H12B—C12—H12C	109.5	O1—Co1—N4	89.95 (11)
N3—C13—C14	122.8 (3)	O3—Co1—N1	89.86 (11)
N3—C13—H13	118.6	O1—Co1—N1	93.03 (11)
C14—C13—H13	118.6	N4—Co1—N1	176.27 (12)
C15—C14—C13	117.4 (3)	O3—Co1—N3	167.52 (11)
C15—C14—C23	123.5 (3)	O1—Co1—N3	98.53 (12)
C13—C14—C23	119.1 (3)	N4—Co1—N3	83.14 (12)
C14—C15—C16	120.8 (3)	N1—Co1—N3	94.19 (12)
C14—C15—H15	119.6	O3—Co1—N2	97.86 (12)
C16—C15—H15	119.6	O1—Co1—N2	166.77 (11)
C15—C16—C17	119.1 (3)	N4—Co1—N2	94.61 (12)
C15—C16—H16	120.4	N1—Co1—N2	82.98 (12)
C17—C16—H16	120.4	N3—Co1—N2	94.34 (12)
N3—C17—C16	120.6 (3)	O3—Co1—C25	34.81 (12)
N3—C17—C18	114.8 (3)	O1—Co1—C25	34.63 (12)
C16—C17—C18	124.6 (3)	N4—Co1—C25	91.91 (13)
N4—C18—C19	120.8 (3)	N1—Co1—C25	91.82 (12)
N4—C18—C17	113.6 (3)	N3—Co1—C25	133.08 (13)
C19—C18—C17	125.6 (3)	N2—Co1—C25	132.58 (13)
C20—C19—C18	120.1 (4)	C25—O1—Co1	90.3 (2)
C20—C19—H19	120.0	C25—O3—Co1	90.2 (2)
C18—C19—H19	120.0		
			
N1—C1—C2—C3	0.1 (6)	C22—N4—Co1—N3	179.6 (3)
N1—C1—C2—C11	−178.4 (3)	C18—N4—Co1—N3	0.0 (2)
C1—C2—C3—C4	0.8 (6)	C22—N4—Co1—N2	85.7 (3)
C11—C2—C3—C4	179.3 (4)	C18—N4—Co1—N2	−93.9 (3)
C2—C3—C4—C5	−1.1 (6)	C22—N4—Co1—C25	−47.3 (3)
C3—C4—C5—N1	0.5 (5)	C18—N4—Co1—C25	133.2 (3)
C3—C4—C5—C6	−178.4 (3)	C1—N1—Co1—O3	−80.4 (3)
N1—C5—C6—N2	−1.9 (4)	C5—N1—Co1—O3	97.7 (2)
C4—C5—C6—N2	177.0 (3)	C1—N1—Co1—O1	−11.0 (3)
N1—C5—C6—C7	179.3 (3)	C5—N1—Co1—O1	167.1 (2)
C4—C5—C6—C7	−1.8 (6)	C1—N1—Co1—N3	87.8 (3)
N2—C6—C7—C8	−1.5 (5)	C5—N1—Co1—N3	−94.2 (2)
C5—C6—C7—C8	177.2 (3)	C1—N1—Co1—N2	−178.3 (3)
C6—C7—C8—C9	−0.8 (6)	C5—N1—Co1—N2	−0.3 (2)
C7—C8—C9—C10	1.4 (5)	C1—N1—Co1—C25	−45.6 (3)
C7—C8—C9—C12	−177.3 (4)	C5—N1—Co1—C25	132.4 (2)
C8—C9—C10—N2	0.1 (5)	C13—N3—Co1—O3	103.1 (5)
C12—C9—C10—N2	178.9 (3)	C17—N3—Co1—O3	−73.0 (6)
N3—C13—C14—C15	−0.1 (5)	C13—N3—Co1—O1	88.1 (3)
N3—C13—C14—C23	179.2 (3)	C17—N3—Co1—O1	−88.0 (2)
C13—C14—C15—C16	1.1 (5)	C13—N3—Co1—N4	177.0 (3)
C23—C14—C15—C16	−178.3 (4)	C17—N3—Co1—N4	0.9 (2)
C14—C15—C16—C17	−0.7 (5)	C13—N3—Co1—N1	−5.6 (3)
C15—C16—C17—N3	−0.6 (5)	C17—N3—Co1—N1	178.3 (2)
C15—C16—C17—C18	178.9 (3)	C13—N3—Co1—N2	−88.8 (3)
N3—C17—C18—N4	1.6 (4)	C17—N3—Co1—N2	95.1 (2)
C16—C17—C18—N4	−178.0 (3)	C13—N3—Co1—C25	90.9 (3)
N3—C17—C18—C19	−178.2 (3)	C17—N3—Co1—C25	−85.3 (3)
C16—C17—C18—C19	2.3 (6)	C10—N2—Co1—O3	87.5 (3)
N4—C18—C19—C20	0.7 (5)	C6—N2—Co1—O3	−89.7 (2)
C17—C18—C19—C20	−179.5 (3)	C10—N2—Co1—O1	103.4 (5)
C18—C19—C20—C21	0.0 (6)	C6—N2—Co1—O1	−73.9 (6)
C19—C20—C21—C22	−0.8 (5)	C10—N2—Co1—N4	−6.4 (3)
C19—C20—C21—C24	178.5 (4)	C6—N2—Co1—N4	176.3 (2)
C20—C21—C22—N4	0.9 (6)	C10—N2—Co1—N1	176.4 (3)
C24—C21—C22—N4	−178.5 (3)	C6—N2—Co1—N1	−0.8 (2)
C2—C1—N1—C5	−0.7 (5)	C10—N2—Co1—N3	−89.9 (3)
C2—C1—N1—Co1	177.2 (3)	C6—N2—Co1—N3	92.9 (2)
C4—C5—N1—C1	0.5 (5)	C10—N2—Co1—C25	90.4 (3)
C6—C5—N1—C1	179.4 (3)	C6—N2—Co1—C25	−86.8 (3)
C4—C5—N1—Co1	−177.7 (3)	O1—C25—Co1—O3	179.8 (3)
C6—C5—N1—Co1	1.3 (4)	O3—C25—Co1—O1	−179.8 (3)
C9—C10—N2—C6	−2.4 (5)	O1—C25—Co1—N4	−87.1 (2)
C9—C10—N2—Co1	−179.5 (3)	O3—C25—Co1—N4	93.0 (2)
C7—C6—N2—C10	3.0 (5)	O1—C25—Co1—N1	92.7 (2)
C5—C6—N2—C10	−175.8 (3)	O3—C25—Co1—N1	−87.1 (2)
C7—C6—N2—Co1	−179.5 (3)	O1—C25—Co1—N3	−4.8 (3)
C5—C6—N2—Co1	1.7 (4)	O3—C25—Co1—N3	175.40 (17)
C14—C13—N3—C17	−1.2 (5)	O1—C25—Co1—N2	174.82 (18)
C14—C13—N3—Co1	−177.1 (3)	O3—C25—Co1—N2	−5.0 (3)
C16—C17—N3—C13	1.5 (5)	O2—C25—O1—Co1	179.6 (4)
C18—C17—N3—C13	−178.0 (3)	O3—C25—O1—Co1	0.1 (3)
C16—C17—N3—Co1	178.0 (3)	O3—Co1—O1—C25	−0.1 (2)
C18—C17—N3—Co1	−1.6 (4)	N4—Co1—O1—C25	93.4 (2)
C21—C22—N4—C18	−0.2 (5)	N1—Co1—O1—C25	−88.8 (2)
C21—C22—N4—Co1	−179.7 (3)	N3—Co1—O1—C25	176.5 (2)
C19—C18—N4—C22	−0.7 (5)	N2—Co1—O1—C25	−16.9 (6)
C17—C18—N4—C22	179.6 (3)	O2—C25—O3—Co1	−179.6 (4)
C19—C18—N4—Co1	178.9 (3)	O1—C25—O3—Co1	−0.1 (3)
C17—C18—N4—Co1	−0.8 (4)	O1—Co1—O3—C25	0.1 (2)
C22—N4—Co1—O3	−12.4 (3)	N4—Co1—O3—C25	−88.6 (2)
C18—N4—Co1—O3	168.0 (2)	N1—Co1—O3—C25	93.4 (2)
C22—N4—Co1—O1	−81.8 (3)	N3—Co1—O3—C25	−15.7 (6)
C18—N4—Co1—O1	98.6 (2)	N2—Co1—O3—C25	176.3 (2)

### Hydrogen-bond geometry (Å, º)

**Table:**

*D*—H···*A*	*D*—H	H···*A*	*D*···*A*	*D*—H···*A*
C1—H1···O1	0.93	2.43	2.931 (5)	114
C3—H3···Br1^i^	0.93	2.80	3.718 (4)	168
C4—H4···O1^ii^	0.93	2.51	3.257 (4)	138
C10—H10···N4	0.93	2.53	3.036 (4)	114
C11—H11*A*···Br1^iii^	0.96	2.91	3.810 (4)	157
C13—H13···N1	0.93	2.52	3.023 (4)	114
C19—H19···O3^iv^	0.93	2.52	3.284 (4)	140
C20—H20···Br1^iv^	0.93	2.85	3.778 (4)	172
C22—H22···O3	0.93	2.44	2.939 (4)	113
C23—H23*B*···O4^v^	0.96	2.42	3.330 (6)	158
C24—H24*A*···Br1	0.96	2.93	3.836 (5)	158

Symmetry codes: (i) *x*−1, −*y*+1/2, *z*−1/2; (ii) *x*, −*y*+1/2, *z*−1/2; (iii) *x*−1, *y*, *z*; (iv) *x*, −*y*+1/2, *z*+1/2; (v) −*x*+1, *y*−1/2, −*z*+3/2.
